# Reactive Oxygen Species and Nuclear Factor Erythroid 2-Related Factor 2 Activation in Diabetic Nephropathy: A Hidden Target

**DOI:** 10.4172/2155-6156.1000547

**Published:** 2015-05-10

**Authors:** Shaaban Abdo, Shao-Ling Zhang, John S.D. Chan

**Affiliations:** Department of Medicine, University of Montreal and Research Center Hospital of QC, Canada

**Keywords:** Nrf2, Angiotensinogen expression, Renin-angiotensin system

## Abstract

Hyperglycemia, oxidative stress and renin-angiotensin system (RAS) dysfunction have been implicated in diabetic nephropathy (DN) progression, but the underlying molecular mechanisms are far from being fully understood. In addition to the systemic RAS, the existence of a local intrarenal RAS in renal proximal tubular cells has been recognized. Angiotensinogen is the sole precursor of all angiotensins (Ang). Intrarenal reactive oxygen species (ROS) generation, Ang II level and RAS gene expression are up-regulated in diabetes, indicating that intrarenal ROS and RAS activation play an important role in DN. The nuclear factor erythroid 2-related factor 2 (Nrf2)-Kelch-like ECH-associated protein 1 (Keap1) pathway is one of the major protective processes that occurs in response to intracellular oxidative stress. Nrf2 stimulates an array of antioxidant enzymes that convert excessive ROS to less reactive or less damaging forms. Recent studies have, however, revealed that Nrf2 activation might have other undesirable effects in diabetic animals and in diabetic patients with chronic kidney disease. This mini-review summarizes current knowledge of the relationship between ROS, Nrf2 and intra renal RAS activation in DN progression as well as possible novel target(s) for DN treatment.

## Introduction

Diabetes is an epidemic disease that is imposing a heavy healthcare burden globally. Its incidence is continuing to rise unabated. According to recent estimates by the International Diabetes Federation, the number of people with diabetes will surge from 382 to 592 million in less than 25 years. Diabetes and its associated complications caused 5.1 million deaths in 2013 [1]. Diabetic nephropathy (DN), a clinical syndrome, is the result of gluco-lipotoxicity. It impacts the kidneys, eliciting progressive renal insufficiency, persistent albuminuria, hypertension and decreased glomerular filtration rate (GFR) [2]. DN affects approximately one-third of people with type 1 or type 2 diabetes mellitus (T1D and T2D respectively) [3]. It is the most common cause of end-stage renal disease (ESRD) in the West and possibly throughout the world, accounting for more than 50% of all patients with ESRD. Although insulin and oral anti-diabetic drugs, along with diet and exercise, are the cornerstones of diabetes mellitus and DN treatment [4,5], their underlying mechanisms remain incompletely understood.

## The Renin-Angiotensin System (RAS)

The RAS is a hormonal system that regulates sodium balance, body fluid homeostasis and arterial pressure [6]. All RAS components have been identified in the kidneys [7], including mRNA and protein of angiotensinogen (Agt), renin, angiotensin-converting enzyme (ACE), angiotensin-converting enzyme 2, angiotensin II (Ang II) receptor subtypes 1 and 2 (AT1R and AT2R) as well as Ang 1–7 receptor (MasR). In the kidneys, Agt is expressed predominantly in renal proximal tubular cells (RPTCs): it is converted into inactive Ang I by renin and then into biologically-active Ang II by ACE. Ang II could be further cleaved to Ang 1–7. Intrarenal RAS gene expression is elevated in diabetes [8], strongly indicating that intrarenal RAS activation plays a significant role in DN progression including interstitial tubule-fibrosis and tubular atrophy [9].

Hypertension usually accompanies diabetes mellitus, which increases kidney damage when normo-albuminuria evolves to macro-albuminuria [10]. Ang II, the main product of RAS activation, contributes to various physiological and pathological renal and cardiovascular mechanisms through AT1R stimulation [11]. It has been reported that early streptozotocin (STZ)-induced diabetes down-regulates rat kidney AT2R and increases AT1R [12]. Moreover, several studies have shown that AT2R expression is augmented by insulin treatment [13] and down-regulated by Ang II infusion or epidermal growth factor treatment [14]. Arresting the effects of RAS activation with either ACE inhibitors [15] to decrease Ang II production or with angiotensin-receptor blockers (ARBs) to block AT1R activation, leads to lower intraglomerular pressure [16], reduced systemic hypertension and, consequently, decreased renal interstitial fibrosis.

## Reactive Oxygen Species (ROS)

Living organisms produce ROS as a result of normal cellular metabolism. ROS steady-state levels are required for cell proliferation, differentiation [17] and degradation of misfolded/damaged proteins by ubiquitin and 26S proteasome [18]. In contrast, excessive ROS production damages cellular components, such as DNA, proteins and lipids [19]. Superoxide (O2•−), hydrogen peroxide (H2O2) and nitric oxide are free radicals essential for normal physiological development, but also mediate cellular damage in disease states [20]. Cellular sources of ROS production include plasma membrane nicotinamide adenine dinucleotide phosphate oxidase (NADPH oxidase), intracellular cytosolic xanthine oxidase, peroxisomal oxidases, endoplasmic reticular oxidases and mitochondrial electron transport constituents [19]. Mitochondrial electron transport of aerobic respiration, considered to be the main source of ROS, has been implicated in many disorders [21,22]. It has been estimated that 0.2 to 2% of the oxygen consumed by mitochondria is reduced to O2•− [23].

Oxidative stress occurs in cellular systems when the production of free radical moieties exceeds the antioxidant capacity of those systems, evoking a shift in balance between oxidants/antioxidants in favour of oxidants [24]. In certain pathological conditions, increased generation of ROS and/or antioxidant defence system depletion generate enhanced ROS activity and oxidative stress, resulting in tissue damage. Several systemic diseases, such as hypertension, diabetes mellitus, metabolic syndrome and infections, induce renal oxidative stress [25]. Excessive ROS production in the kidneys has been reported in different hypertensive animal models [26,27], including Ang II-induced hypertensive rats [28], N-omega-nitro-L-arginine-induced hypertensive rats [29], Dahl salt-sensitive hypertensive rats [30] and spontaneously hypertensive rats [31]

## Nrf2 Activation and Redox Balance

Oxidative stress is the most common cause of tissue injury. In order to maintain redox homeostasis balance to prevent cellular damage, an intracellular antioxidant system, i.e., Nuclear factor erythroid 2-related factor 1–3 (Nrf 1–3), is activated to detoxify the potential harmful substance. Nrf1, Nrf2 and Nrf3 genes encode a member of the cap ‘n’ collar basic-region leucine zipper family, which is vital in regulating antioxidant gene expression [32], development and redox balance [33]. Nrf1 is an important player in redox balance during development [34]. Homozygous Nrf1 null mice die in utero [35], while Nrf2- and Nrf3-null mice develop normally with no obvious phenotypic differences compared to wild type controls [36,37]. Nrf2-deficient female mice develop lupus-like autoimmune nephritis [38]. Nrf1 and Nrf2 deficiency, however, has been shown to culminate in early embryonic lethality and severe oxidative stress [39]. Nrf2 is a transcriptional factor consisting six evolutionarily highly conserved domains, Neh1–6 [40] that activates the transcription of an array of antioxidant genes [41]. Several reports showed that Nrf2 activation may protect against human disease such as cardiovascular disease [42], cancer [43], neurodegenerative diseases [44] and chronic kidney disease [45].

## Regulation of Nrf2 activation

Nrf2 activation is predominantly regulated by it cytosolic partner, Nrf2 adaptor or Kelch-like ECH-associated protein 1 (Keap1). Keap1 is a protein of five domains: two protein protein interaction motifs, the Kelch domain, the intervening region (IVR), and the BTB domain [46]. Keap1 acts as a cytosolic repressor for Nrf2 in the cytoplasm. Several models have been proposed to understand the mechanism of Nrf2 activation. Under normal physiological conditions, the BTB domain of Keap1 interacts with the Neh2 domain of cytoplasmic Nrf2 and forms complexes with Cullin 3-based E3 ligase [47]. These complexes promote Nrf2 degradation via the ubiquitin proteasome system [48]. In response to oxidative stress or chemo-preventive compounds, Nrf2 dissociates from Keap1 and translocates to the nucleus [49]. However, if Keap 1 binds to other proteins, Nrf2 does not ubiquitinated but activated in a non-canonical, cysteine-independent manner. For example, Lau et al. [50] demonstrated that deregulation of autophagy pathway causes the accumulation of p62 (an autophagic protein) that directly interacts with Keap1, resulting in the inhibition of Keap1-mediated Nrf2 ubiquitination. In addition, Nrf2 has been shown to be regulated at transcriptional level, independent of the Keap1 mechanism. Studies by Kawak et al [51] demonstrated that Nrf2 undergoes autoregulation: Nrf2 binds to its own promoter and induce its own transcriptional activity. Post-translation modifications also play a significant role in Nrf2 function and localization. For example, Nrf2 phosphorylation by protein kinase C at serine 40 residue is critical signaling event to regulate cellular antioxidant response [52]. Furthermore, acetylation-deacetylation of the Nrf2 regulates its transcriptional activity and nucleocytoplasmic localization [53]. Natural or synthetic chemopreventive agents, such as curcumin, flavonoids, oltipraz, butylatedhydroxyanisole, and bardoxolone methyl stimulate Nrf2 activation and nuclear translocation. Within the nucleus, Nrf2 forms a heterodimer with Maf protein and bind to regulatory sequences in the promoter region of various genes, known as antioxidant response elements (AREs). A series of antioxidant and cellular protective genes containing AREs in their promoters have been identified; such as glutathione peroxidase, superoxide dismutase, catalase (Cat), heme-oxygenase, NADPH-quinoneoxidoreductase 1 and glutamate-cysteine ligase [54,55]. Moreover, it has been found that activation of Nrf2 signaling attenuates NFkappaB-inflammatory response and elicits apoptosis [56].

Recent studies, however, revealed that Nrf2 may also affect none-oxidant genes expression. For example, Pendyala et al. [57] demonstrated in human lung endothelium, by chromatin-immunoprecipitation assay, that Nrf2 binds to ARE regions in NADPH oxidase-4 (Nox4) gene promoter and up-regulates its activity. NADPH oxidase-1, -2 and -4 (Nox1, Nox2 and Nox4) have also been shown to be expressed in the renal cortex and Nox4 is the most common isoform expressed in the kidneys [58,59]. Nox4 contributes to basal ROS production through its constitutive activity and increases ROS generation when stimulated by Ang II, glucose, and growth factors [60–62]. Our study in the renal proximal tubules (RPTCs) of diabetic Akita mice (a murine model of T1D) has disclosed that Nrf2 translocates to the nucleus with markedly enhanced NADPH oxidase activity as well as Nox4 mRNA expression compared to non-Akita mice, suggesting that Nrf2 induction of Nox4 expression and activity might be responsible, at least in part, for elevated ROS levels [63]. Intriguingly, these changes are normalized by overexpressing catalase (Cat) in RPTCs of Akita mice, lessening Nrf2 translocation to the nucleus [63]. These observations indicated that constitutive Nrf2 accumulation does not necessary induce absolute protection from environmental influences. Indeed, study has shown that hyperoxia stimulates Nrf2 translocation to the nucleus, and Nrf2 knockdown of Nrf2 gene expression by small interfering RNA (siRNA) attenuates hyperoxia-induced Nox4 expression, suggesting that Nrf2 may up-regulates Nox4 gene expression in certain physiological conditions [57].

## Nrf2 expression in diabetic kidneys

Many studies demonstrated a beneficial effect of Nrf2 activation in none-diabetic kidney. Shelton et al. [64] reported that Nrf2 activation protects the kidney against oxidative stress that results from ischemic-reperfusion [65,66], renal injury caused by excessive heavy metals [67,68], and ameliorate cyclosporine A nephrotoxicity [69].

In animals with streptozotocin (STZ)-induced diabetes, Nrf2 ablation appears to worsen inflammation, oxidative stress, and nephropathy [70,71]. Moreover, dietary supplementation with sulforaphane or cinnamic aldehyde (Nrf2 activators) reduces albuminuria and renal oxidative damage in STZ-induced diabetic mice [71,72]. It also improves glucose control, lowers plasma triglyceride and FFA levels, decreases hepatic lipid accumulation and inflammation in both high-fat diet-induced and genetic mouse models of obesity and diabetes [73,74]. Thus, Nrf2 appears to be cytoprotective in none-diabetic and diabetic animal models.

## Nrf2 activation in human chronic kidney disease (CKD)

A phase II trial of bardoxolone methyl (a Nrf2 activator) involving patients with advanced CKD and diabetes was carried out based on compelling evidence of the association between oxidative stress, inflammation and DN progression. Pergola et al. [75] initiated a small clinical trial of 227 T2D patients with moderate to severe CKD (BEAM study) and observed that bardoxolone methyl treatment increased estimated GFR in a dose-dependent manner in patients at 24 weeks with persistence up to 52 weeks. These encouraging data motivated de Zeeuw et al. [76] to investigate the effect of bardoxolone methyl in a phase III clinical trial in 2,185 patients from different countries with stage III CKD and T2D (BEACON Study). However, the trial was terminated early in October 2012 because bardoxolone methyl treatment neither improved renal function nor reduced the risk of ESRD, but was actually associated with increased risk of cardiovascular death. In fact, bardoxolone methyl-treated patients presented significant increment of estimated GFR, blood pressure (BP) and urinary albumin-to-creatinine ratio, with decreased body weight in comparison to the placebo group, and acquired a higher risk of cardiovascular events.

**Figure 1:**
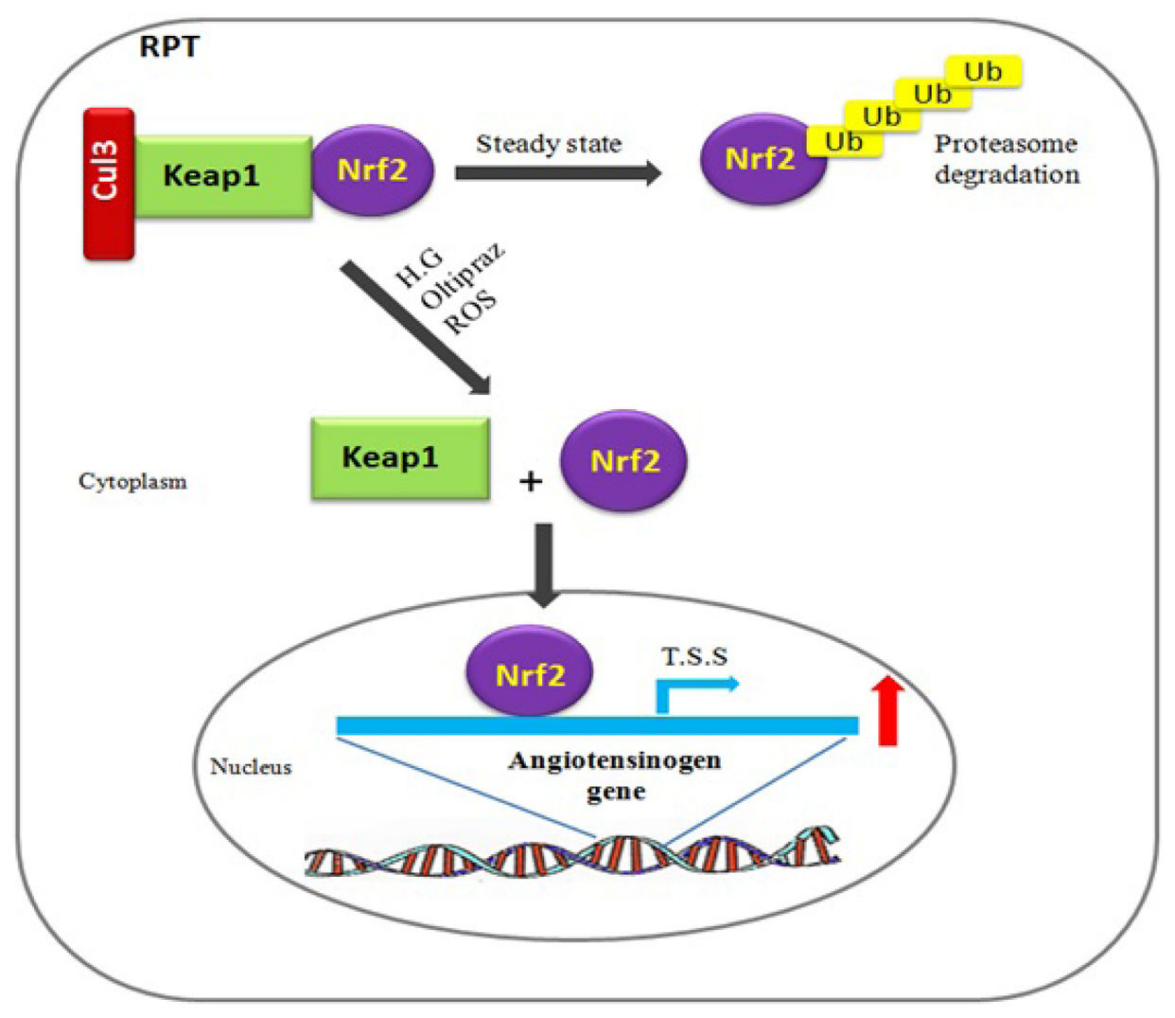
Proposed mechanism of Nrf2 activation on Agt gene expression in RPTs: Under normal conditions, Nrf2 is bound to Keap1 in the cytoplasm and is subject to ubiquitination and proteasome degradation. Upon exposure to high glucose (HG), oxidative stressor or Nrf2 activators (oltipraz), Nrf2-Keap1 complexes dissociate and Nrf2 translocates to the nucleus. Nrf2 binds to Nrf2-REs in the Agt gene promoter, stimulating Agt gene transcription and intrarenal RAS activation, and leading to the development of hypertension and nephropathy in diabetes. TSS (transcriptional start site)

Studies were performed in Zucker diabetic fatty (ZDF) rats, a T2D model, to confirm the potential effects of bardoxolone methyl in DN. Zoja et al. [77] reported that ZDF rats receiving the bardoxolone methyl analogue RTA 405 presented DN deterioration impacting the liver. In contrast, a study of RTA 405 and dh404 sponsored by Reata Pharmaceuticals via Biomodels LLC in ZDF rats showed heightened urinary albumin/creatinine ratios with both compounds, but no adverse effects in the liver [78]. Inconsistencies between these two studies were observed. Subsequent analysis by Reata Pharmaceuticals revealed the presence of unknown impurities in RTA 405 that they had supplied for both studies. The concentrations of these impurities were very low, indicating that they might have been extremely toxic. Unfortunately, no further work on ‘pure’ RTA 405 has been reported. Whether Nrf2 activation worsens human CKD progression remains uncertain. Despite such conflicting results, there is still evidence that Nrf2-dependent events are protective against DN progression [70,71,73,74]. These findings led us to investigate whether Nrf2 activator treatment would have adverse side-effects in the kidneys by itself or whether Nrf2 pathway activation would elevate BP and cause kidney injury. To answer this question, we explored the relationship between renal Nrf2 activation and Agt gene expression in the RPTs of wild type mice since we previously reported that high glucose stimulated Agt gene expression via ROS generation [79] and that transgenic mice overexpressing Agt in their RPTCs exhibited hypertension, albuminuria and renal injury [80]. Indeed, we found that oltipraz (a Nrf2 activator) administration heightened both Nrf2 and renal Agt gene expression. Moreover, deletion of Nrf2-response element (RE) sites in the Agt promoter prevented oltipraz stimulation of Agt gene transcription in RPTCs [63]. These observations strongly suggest that Nrf2 may play dual roles, with stimulation of both antioxidant and hypertensinogenic genes, such as Agt, as illustrated in the schematic Figure 1 below:

In conclusion, clinical studies have shown that Nrf2 activation by bardoxolonemethy increases systemic hypertension aggravates urinary albumin excretion and augments the risk of cardiovascular death in type II diabetic patients with advanced CKD [76]. These observations suggest that Nrf2 over-activation may not always exhibit beneficial in diabetes. Hyperglycemia-induction of ROS generation and chronic activation of Nrf2 signaling may induce intrarenal RAS activation, lending to systemic hypertension, albuminuria and tubular apoptosis and atrophy, further aggravate nephropathy progression. Thus, blocking chronic Nrf2 activation in diabetic kidneys would be a novel therapeutic target for DN treatment. Indeed, the recent study by Tan et al [81] demonstrated that low doses of dh404 lessened and high doses worsened diabetes-associated atherosclerosis and kidney disease in STZ-induced diabetic apoE−/− mice as well as the study by Vaziri et al. [82] demonstrated that in diabetic obese Zucker rats, low doses of dh404 restore Nrf2 activity and ameliorate kidney injury whereas high doses of dh404 reinforce proteinuria, renal dysfunction and histological abnormalities. These observations strongly suggest the possible dual function of Nrf2 activation in the diabetic kidney, depending on the level of Nrf2 activation in diabetes.

## Abbreviations

ACE: Angiotensin-Converting Enzyme
Agt: Angiotensinogen
Ang II: Angiotensin II
Ang 1–7: Angiotensin 1–7
ARE: Antioxidant Responsive Element
AT1R: Ang II subtype 1 receptor
AT2R: Ang II subtype 2 receptor
BP: Blood Pressure
Cat: Catalase
CKD: Chronic Kidney Disease
DN: Diabetic Nephropathy
ESRD: End Stage Renal Disease
GFR: Glomerular Filtration Rate
HG: High Glucose
hnRNP F and hnRNP K: Heterogeneous Nuclear Ribonucleoproteins F and K
H2O2: Hydrogen Peroxide
Keap1: Kelch-like ECH-associated Protein 1
NADPH oxidase: Nicotinamide Adenine Dinucleotide Phosphate Oxidase
Nrf2: Nuclear Factor Erythroid 2-Related Factor 2
O2•−: Superoxide
RAS: Renin-Angiotensin System
Res: Responsive Elements
ROS: Reactive Oxygen Species
RPTs: Renal Proximal Tubules
Runx2: Runt-Related Transcription Factor 2
RPTCs: Renal Proximal Tubular Cells
STZ: Streptozotocin
T1D and T2D: Type 1 And Type 2 Diabetes Mellitus
ZDF: zucker diabetic fatty rats
